# Degradation of IRAK4 for the treatment of lipopolysaccharide-induced acute lung injury in mice

**DOI:** 10.3389/fphar.2025.1609923

**Published:** 2025-07-23

**Authors:** Bijun Ye, Shiyan Chen, Xiaohao Huang, Yinhong Qiu, Ruixiang Luo, Lulu Zheng

**Affiliations:** ^1^Department of Neurology, Tiantai People’s Hospital of Zhejiang Province (Tiantai Branch of Zhejiang Provincial People’s Hospital), Hangzhou Medical College, Hangzhou, Zhejiang, China; ^2^Department of Pharmacy, Tongde Hospital of Zhejiang Province, Hangzhou, Zhejiang, China

**Keywords:** interleukin-1 receptor-associated kinase 4 (IRAK4), acute lung injury, KT-474, inflammatory disease, anti-inflammation

## Abstract

Excessive pulmonary inflammation in acute lung injury (ALI) results in high patient mortality. Interleukin-1 receptor-associated kinase 4 (IRAK4) is a potential therapeutic target for inflammatory diseases. However, due to the dual functionality of IRAK4 as both an active kinase and a scaffolding protein, inhibiting its kinase activity yield moderate anti-inflammatory results. The present study explored the efficacy of KT-474, a prototypical IRAK4 degrader, which effectively diminished cellular IRAK4 levels, achieving half-maximal degradation at a concentration of 4.0 nM in RAW 264.7 cells. KT-474 effectively inhibited the activation of downstream nuclear factor (NF)-κB signaling, exhibiting stronger pharmacological impacts compared to conventional kinase inhibitors. Additionally, a lipopolysaccharide-induced acute inflammatory mouse model was established, and KT-474 displayed significant therapeutic benefits *in vivo* compared to kinase inhibitors. Therefore, these findings highlight the therapeutic potential of IRAK4 degrader for the treatment of acute lung injury.

## Graphical Abstract

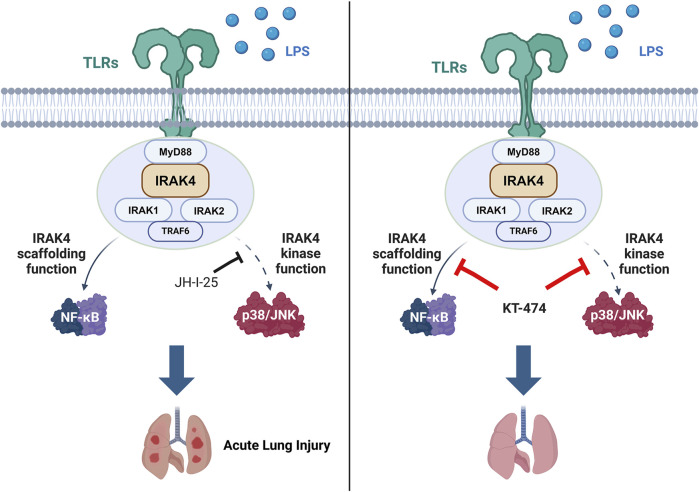

## Highlights

• KT-474 demonstrates preclinical efficacy in acute lung injury
• KT-474 attenuates ALI via blocking both kinase role and scaffolding role of IRAK4
• KT-474 is applicable therapeutics in TLR-induced inflammatory diseases

## 1 Introduction

Acute lung injury (ALI) and its more severe form, acute respiratory distress syndrome (ARDS), are complications of diverse conditions, including systematic inflammation, direct injury, and infections in the lung (Liu et al., 2021). ALI carries a devastatingly high mortality rate; unfortunately, effective and specialized therapeutic drugs to treat ALI are lacking, underscoring the urgency of developing new treatment modalities. Lipopolysaccharide (LPS) is a well-known endotoxin that is highly expressed and secreted into the lung tissues of ALI patients caused by sepsis. LPS induces the activation of the TLR4 signaling pathway, overexpression of inflammation cytokines, inflammatory reaction, and deathly cytokine storm. During ALI, the overreaction of the immune system results in the secretion of cytokines, including TNF-α, IL-6, adhesion molecule type 1 (ICAM) (Beck-Schimmer et al., 2002), and vascular cell adhesion molecule-1 (VCAM) (Gao et al., 2024), increased vascular permeability, and diffuse alveolar injury. Therefore, inhibiting excessive inflammation by blocking the TLR4 signaling pathway represents an important strategy to treat ALI patients.

Toll-like receptors (TLRs) play a pivotal role in the immune system’s defense mechanisms and inflammatory responses, recognizing a variety of molecular patterns associated with tissue damage and microbial pathogens (Chen et al., 2020). Interleukin-1 receptor (IL-1R)-associated kinase 4 (IRAK4) is a serine/threonine kinase that sits at the crossroads of TLR and IL-1R signaling pathways (Gay et al., 2014). IRAK4 is a key player in the TLR/IL-1R-triggered Myeloid differentiation primary response 88 (MyD88)-dependent signaling cascades (Chaudhary et al., 2015). Previous studies have shown that abnormal IRAK4 activity is linked not only to cancer but also to inflammatory diseases, including sepsis, psoriasis, systemic lupus erythematosus, and rheumatoid arthritis (Li, 2008). Considering its critical role, IRAK4 is a promising therapeutic target for managing chronic inflammatory skin conditions (Lavazais et al., 2023). Consequently, strategies to inhibit IRAK4 are being pursued as potential treatments for a range of autoimmune and inflammatory diseases (Chaudhary et al., 2015).

Stimuli from LPS, IRAK4, IRAK1/2, and MyD88 can form a signalosome called “Myddosome”, a complex-induced downstream signal (Lin et al., 2010). Recent studies have reported the dual roles of IRAK4 in the myddosome complex. Although the kinase activity is essential for the recruitment of IRAK1, it is not necessary for the recruitment of IRAK4 to MyD88 (Kawagoe et al., 2007). Inhibition of IRAK4 kinase activity by a small-molecule compound results in a significantly more stabilized myddosome complex (Nardo et al., 2018). In addition, IRAK4 kinase activity is not required for TLR4 signaling; however, the IRAK4 scaffold function is essential for nuclear factor (NF)-κB activation in TLR4-activated macrophages (Pereira et al., 2022). Moreover, the IRAK4 death domain plays a crucial role in receptor proximal signaling by mediating TLR-induced NF-κB activation (Frączek et al., 2008). IRAK4 also exhibits a critical scaffold function in myddosome formation, although its kinase activity is not required for myddosome assembly.

Due to the significance of IRAK4 in the TLR/IL-1R signaling pathway, many inhibitors targeting IRAK4 have been developed to treat cancers and inflammatory diseases. Nonetheless, the representative IRAK4 inhibitor PF-06650833 for clinical rheumatoid arthritis treatment was withdrawn or terminated. Other representative drugs, such as GS-5718 (NCT05165771, NCT04809623) and zabedosertib (Anderson et al., 2024) (also named BAY1834845, NCT05656911) have also been discontinued, withdrawn, or terminated. All these clinical results suggested that the inhibition of IRAK4 alone may not be very effective, demonstrating that the inhibition of IRAK4 kinase activity alone cannot completely block the TLR signaling transduction.

The innovative proteolysis-targeting chimera (PROTAC) technology offers a potential strategy to overcome the limitations of IRAK4 inhibitors by simultaneously modulating both the catalytic and non-catalytic functions of target proteins (Sun et al., 2022). PROTACs are bifunctional molecules that induce the ubiquitination of the protein of interest (POI), promoting its degradation via the ubiquitin-proteasome system (Burslem and Crews, 2020). To date, only a handful of PROTACs have been devised to disrupt the non-catalytic functions of kinases, such as AURORA-A (Adhikari et al., 2020) and focal adhesion kinase (Gao et al., 2020), along with non-kinase targets (Tu et al., 2021; Wu et al., 2022). PROTAC technology can be applied to remove the IRAK4 scaffold from the myddosome, which could inhibit all downstream signaling of TLR, thereby halting the activation of inflammation and production of cytokines. Therefore, PROTAC targeting IRAK4 may be a new druggable candidate for the IRAK4-dependent disease treatment. At present, several IRAK4 PROTACs are being investigated in clinical trials, such as KT-474, a representative oral bioavailable IRAK4 degrader. KT-474 has been developed to treat hidradenitis suppurativa (IL-1 derived (Tzanetakou et al., 2016), NCT06028230) and atopic dermatitis (multiple factors derived (Peng and Novak, 2015), NCT06058156) (Ackerman et al., 2023). However, the efficacy of IRAK4 degrader on TLR4-induced acute inflammation remains unknown.

This research revealed that the inhibition of IRAK4 had a minimal impact on NF-κB activation in LPS-induced macrophages, which is a process primarily driven by IRAK4’s non-enzymatic (scaffold) activity. Therefore, a typical IRAK4 degrader, KT-474, was applied to effectively eradicate both the kinase and scaffold functions of IRAK4, thereby amplifying its anti-inflammatory efficacy both *in vitro* and *in vivo*. Our study offers valuable insights into the connection between inflammation and IRAK4’s non-enzymatic activity. KT-474 can be deployed to disrupt IRAK4’s non-enzymatic activity in both acute and chronic inflammatory conditions. Until now, most PROTACs under clinical trial have been designed for cancer therapy by degrading the androgen (Gao et al., 2022) or estrogen receptor (Hamilton et al., 2022; Schott et al., 2023), or Bruton’s tyrosine kinase (Békés et al., 2022). Our findings present fresh evidence for the potential use of IRAK4 degraders in the treatment of inflammatory diseases.

## 2 Materials and methods

### 2.1 Cell culture

Mouse macrophage RAW 264.7 cell lines were kindly provided by Cell Bank, Chinese Academy of Sciences. RAW 264.7 cells were cultured in Dulbecco’s modified Eagle’s medium (DMEM) (Cat. BC-M-005, Bio-Channel) containing 10% Fetal Bovine Serum (FBS) (Cat. BC-SE-FBS07, Bio-Channel), and 1% penicillin/streptomycin (Cat. BL505A, Biosharp) at 37°C with 5% CO_2_.

### 2.2 Western blot assay

Proteins were harvested from cells using a lysate solution and then centrifuged at 12,000 rpm for 10 min at a temperature of 4°C. The supernatant was collected, and the protein concentration of each sample was determined using the bicinchoninic acid (BCA) method and adjusted to achieve consistent protein concentrations across samples. Subsequently, the proteins were denatured by heating at 100°C for 10 min after mixing with 5x protein loading buffer. The protein samples were then separated by SDS-PAGE and transferred onto polyvinylidene difluoride (PVDF) membranes using a transfer buffer. These membranes were incubated overnight at 4°C with specific antibodies, which were diluted in a solution of 5% BSA in Tris-buffered saline containing 0.1% Tween-20 (TBST). Enhanced chemiluminescence reagents (NcmECL Ultra; Cat. E422-01; Vazyme Biotech Co., Ltd) were used to visualize the bound antibodies, and images were captured using the Vilber Fusion FX system.

The following primary antibodies were used: anti-IκB-α (1:1000, Cat. ET1603-6), anti-NF-κB p65 (1:1000, Cat. ET1603-12), and anti-p38 (1:1000, Cat. ET1702-65) antibodies, which were obtained from HUABIO. The anti-p-p38(Thr180/Tyr182) (1:1000, Cat. #AF4001) and anti-p-IRAK4(Thr345/Ser346) (1:1000, Cat. #DF7567) antibodies were purchased from Affinity Biosciences. The anti-p-NF-κB p65(Ser468) (1:2000, Cat. 82335-1-RR) and anti-IRAK1 (1:1000, Cat. 10478-2-AP) antibodies were purchased from Proteintech. The anti-p-ERK1/2(Thr202/Tyr204) (1:2000, Cat. #4370), anti-ERK1/2 (1:2000, Cat. #4695) and anti-IRAK4 (1:1000, Cat. #4363) antibodies were purchased from Cell Signaling Technology.

### 2.3 RNA extraction and quantitative real-time PCR

Conventional real-time qPCR was used to assess the mRNA levels in RAW 264.7 cells and mouse lung tissues. The following primer sequences were used.

Mouse *Tnfα*: Forward Primer (5′-3′) TGATCCGCGACGTGGAA; Reverse Primer (5′-3′) ACC​GCC​TGG​AGT​TCT​GGA​A.

Mouse *Il6*: Forward Primer (5′-3′) GAG​GAT​ACC​ACT​CCC​AAC​AGA​CC; Reverse Primer (5′-3′) AAG​TGC​ATC​ATC​GTT​GTT​CAT​ACA.

Mouse *Gapdh*: Forward Primer (5′-3′) GGA​GCG​AGA​TCC​CTC​CAA​AAT; Reverse Primer (5′-3′) GGC​TGT​TGT​CAT​ACT​TCT​CAT​GG.

The protocol is similar to previous study as described (Cai et al., 2024).

### 2.4 Detection of TNF-α and IL-6 secretion

The harvested culture medium and bronchoalveolar lavage fluid (BALF) were subjected to analysis for the detection of TNF-α (Cat. #88-7324, ThermoFisher) and IL-6 (Cat. #88–7064, ThermoFisher). This was achieved using ELISA in strict accordance with the manufacturer’s guidelines.

### 2.5 Immunofluorescence analysis

Furthermore, a staining protocol was implemented to monitor the translocation of the NF-κB p65 subunit within cells to assess NF-κB activation. RAW 264.7 cells were fixed with a 2% formaldehyde solution in PBS for 20 min. This was followed by permeabilization using a 0.5% Triton X-100 solution in PBS for 10 min. Subsequently, the cells were blocked with a 5% BSA solution in PBS for an hour.

After fixation, the cells were incubated with anti-NF-κB p65 antibody (1:100) overnight. The cells were then washed three times with PBS and stained with Alexa Fluor 647-conjugated secondary antibodies (1:500, Cat. A0468; Beyotime Biotechnology) for an hour, and with DAPI (Cat. C1002, Beyotime Biotechnology) for 10 min.

Cell imaging was performed using an EVOS M7000 cell imaging system from ThermoFisher. The statistical analysis of colocalization was executed using the colocalization finder plugin in ImageJ.

### 2.6 Animals

C57BL/6 mice, each with an approximate weight of 20 g, were procured from the Experimental Animal Center of Hangzhou Medical College. The mice were housed in a controlled environment with a consistent temperature and a structured light-dark cycle. They were provided with a nutritionally balanced diet and access to fresh water. The welfare of these animals was meticulously upheld, adhering to the rigorous ethical guidelines set forth by the Animal Ethics Committee of Hangzhou Medical College.

### 2.7 *In vivo* LPS-induced acute lung injury (ALI) model

In the described experimental setup, mice were allocated into five distinct groups, with each group comprising eight mice:

I. Control mice (Con);
II. LPS-induced acute lung injury (ALI) mice (LPS; 5 mg/kg).
III. LPS-induced ALI mice treated with 10 mg/kg of KT-474 (LPS +10 mg/kg KT-474);
IV. LPS-induced ALI mice treated with 20 mg/kg of KT-474 (LPS +20 mg/kg KT-474);
V. LPS-induced ALI mice treated with 20 mg/kg of JH-I-25 (LPS + JH-I-25).

For the treatment, KT-474 and JH-I-25 were administered intragastrically at a dosage of 200 μL/20 g body weight. The control and LPS groups received an equivalent volume of vehicle solution, which consisted of 5% DMSO, 30% PEG-400, and 65% saline. Following a 12-h treatment period with the vehicle, KT-474, or JH-I-25, the mice were then challenged with 5 mg/kg of LPS through intratracheal instillation except for the control group. Six hours after LPS administration, the mice were euthanized, and BALF, serum, and major organ tissue samples were collected for subsequent testing. The collected BALF samples were subjected to centrifugation at 1000 rpm for 10 min at a temperature of 4°C. The supernatant was collected and the concentrations of cytokines TNF-α and IL-6 were detected. These cytokines are key markers of inflammation, and their levels in the BALF can provide insight into the efficacy of the treatments in reducing lung inflammation in the context of LPS-induced ALI.

### 2.8 Immunohistochemical determination

The lung tissues were first fixed in 4% paraformaldehyde for a period of 48 h. After fixation, the tissues were dehydrated through a graded series of alcohol concentrations and then embedded in paraffin. Subsequently, 5 µm-thick sections of the paraffin-embedded tissues were prepared for immunohistochemical staining. H&E staining was performed to visualize the tissue morphology and assess histopathological changes. The stained tissue sections were then examined under a light microscope. For a more objective and quantitative assessment, the images were analyzed using ImageJ software. Lung injury was quantified using the Smith scoring method, consistent with previously published studies (Xiao et al., 2025).

For F4/80 and Myeloperoxidase (MPO) staining, lung tissue that was embedded in paraffin was subjected to deparaffinization and rehydration, followed by antigen retrieval using a citrate buffer (pH 6.0) heated to boiling for 10 min. Thereafter, endogenous peroxidase activity was neutralized with a 3% hydrogen peroxide solution. The sections were then exposed to 5% bovine serum albumin (BSA) for 1 h to block non-specific binding, and then incubated with the F4/80 or MPO primary antibodies for 12 h on a shaker maintained at 4°C. The sections were treated with an HRP-conjugated secondary antibody for 15 min, developed with DAB chromogen, and counterstained with hematoxylin. The samples were observed under an inverted fluorescent microscope. The intensity of the histochemical staining was quantitatively analyzed using ImageJ software (NIH).

### 2.9 Statistical analysis

The data presented in this study was collected from a minimum of three independent trials and was expressed as the mean ± standard error of the mean (SEM). Statistical analysis was conducted using GraphPad Prism (GraphPad).

For comparisons between two sets of data, a two-tailed unpaired Student’s t-test was employed. When more than two data groups were compared, one-way ANOVA followed by Dunnett’s *post hoc* test was used. The colocalization level of the NF-κB p65 subunit was calculated for each individually transfected cell using the ImageJ colocalization finder plugin, with the Pearson value indicating the degree of colocalization.

The threshold for statistical significance was set at *P* < 0.05. Post-tests were only conducted if the F statistic achieved a P-value less than 0.05, assuming the absence of significant discrepancies in variance homogeneity.

## 3 Result

### 3.1 IRAK4 is overexpressed in ALI mice model

**FIGURE 1:**
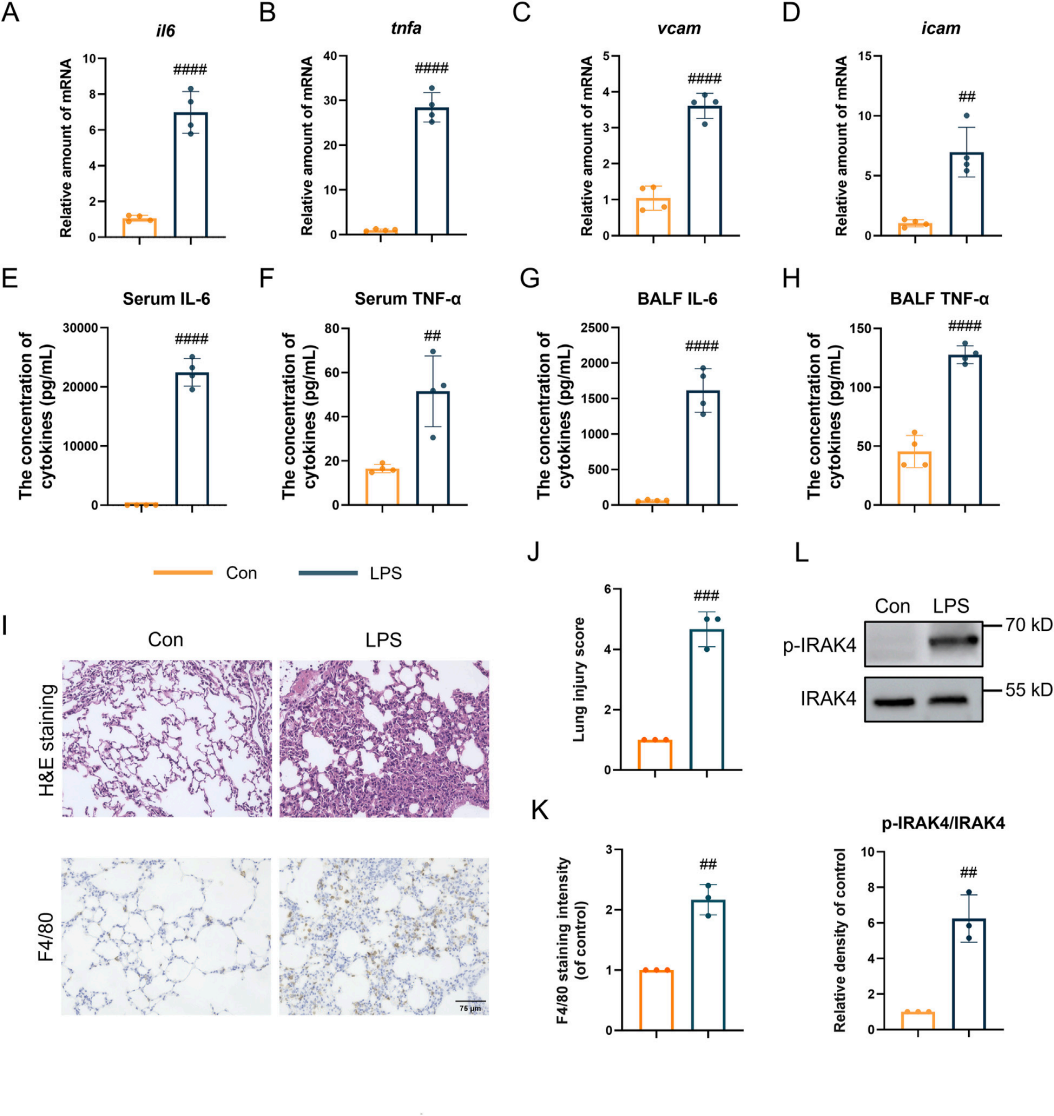
IRAK4 is overexpressed in ALI mice model. **(A–D)** *Il6* **(A)**, *Tnfα* **(B)**, *Vcam* **(C)**, *Icam* **(D)** levels in ALI lung tissue measured by real-time polymerase chain reaction (PCR). **(E,F)** Serum IL-6 **(E)** and TNFα **(F)** level measured by ELISA. **(G,H)** IL-6 and TNFα level in BALF measured by ELISA. **(I)** Representative H&E staining and F4/80 immunohistochemical images of mice lung tissue. **(J)** Smith scoring analysis of **(I)**. **(K)** F4/80 staining intensity analysis by ImageJ in lower **(I)**. **(L)** Representative immunoblots and intensity analysis of lung tissue. Scale bar, 75 μm. Student’s t-test for comparisons of differences in the means of each group. n = 3-4 per group; mean ± SD; **P < 0.01, ***P < 0.001, and ****P < 0.0001; n.s. = not significant.

Considering the critical function of IRAK4, the phosphorylation of IRAK4 was evaluated in an LPS-induced acute lung injury (ALI) model. According to the data in Figures 1A–D, the gene expression of pro-inflammatory markers *Il6*, *Tnfα*, *Vcam*, and *Icam* was found to be upregulated in the ALI model. Additionally, LPS-induced ALI mice exhibited elevated levels of TNF-α and IL-6 in both serum and BALF (Figures 1E–H). Histopathological analysis of hematoxylin and eosin (HE)-stained lung tissues (upper Figures 1I,J) revealed erythrocyte accumulation in the pulmonary capillaries, thickening of the pulmonary septum, fibrin exudation in the alveolar spaces, and disrupted alveolar architecture, confirming the successful induction of an inflammatory response by LPS. Moreover, immunohistochemistry analysis revealed an accumulation of macrophages in the lungs, as indicated by an increase in F4/80, a specific macrophage marker, suggesting the occurrence of an inflammatory cascade (lower Figures 1I,K). Significantly, IRAK4 phosphorylation was found to be significantly elevated in tissue samples induced by ALI (Figure 1L), consistent with its critical role in the LPS-stimulated TLR4 signaling pathway.

### 3.2 KT-474 blocks the NF-κB and MAPK signaling pathway activation in RAW 264.7 cells

**FIGURE 2:**
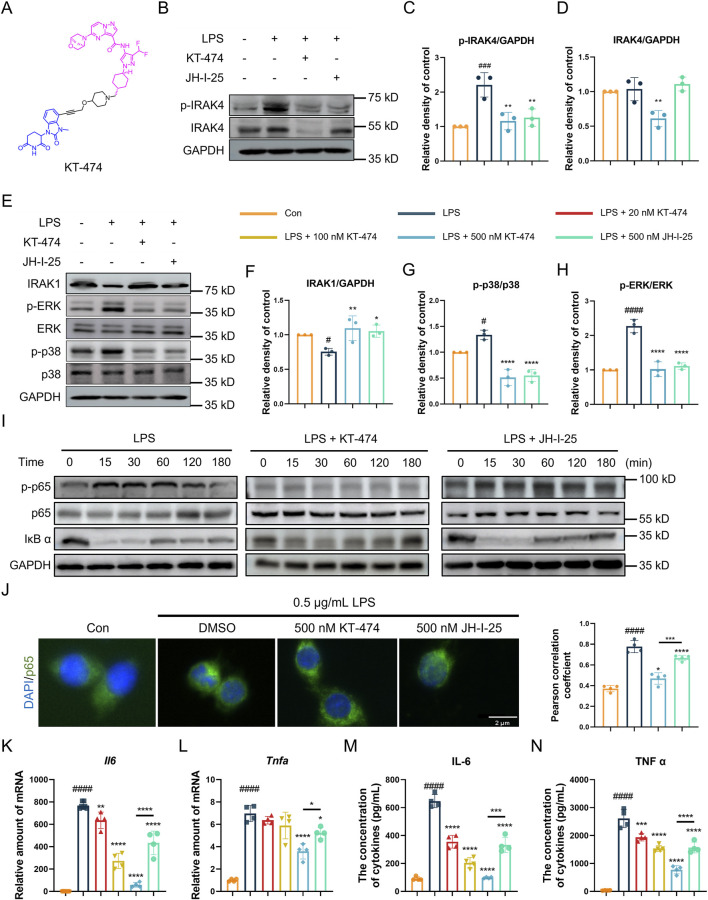
KT-474 blocks both the kinase and scaffolding functions of IRAK4. **(A)** Chemical structure of KT-474. **(B)** Immunoblotting analysis of p-IRAK, IRAK4 and GAPDH in RAW 264.7 cells pretreated with 0.5 μM KT-474 and JH-I-25 for 24 h and stimulated with 0.5 μg/mL LPS for 2 h **(C,D)** Statistical analysis of **(B)**. **(E)** Immunoblotting analysis of IRAK1, p-ERK, ERK, p-p38, p38 and GAPDH in RAW 264.7 cells treated as **(B)**. **(F–H)** Statistical analysis of **(E)**. **(I)** Immunoblotting analysis of NF-κB pathway activation in RAW 264.7 cells within 15, 30, 60, 120, and 180 min. **(J)** Representative immunofluorescence images and colocalization analysis of RAW 264.7 cells stained with DAPI (blue) and p65 (green). **(K,L)** Real-time polymerase chain reaction (PCR) analysis of *Il6* **(K)** and *Tnfα* **(L)** levels in the RAW 264.7 cell lysate. **(M,N**) Enzyme-linked immunosorbent assay (ELISA) of IL-6 **(M)** and TNF-α **(N)** levels in RAW 264.7 cells treated as panel **(D)**. Scale bar, 2 μm. One-way ANOVA with Dunnett’s *post hoc* test for multiple comparisons of differences in the means of each group. n = 3-4 per group; mean ± SD; *P < 0.05, **P < 0.01, ***P < 0.001, and ****P < 0.0001; n.s. = not significant.

Based on the function of IRAK4 in the LPS-induced acute lung injury model, the efficacy of KT-474 (structure shown in Figure 2A) in suppressing inflammatory reactions was further explored *in vitro*. RAW 264.7 cells were pre-incubated with 500 nM KT-474 or a representative IRAK4 inhibitor JH-I-25 and stimulated with 0.5 μg/mL LPS for 1 h. At this concentration, neither compound exhibited significant cytotoxicity, ensuring that the observed effects were not confounded by cell viability issues (Supplementary Figure S1). Western blot analysis revealed that both KT-474 and JH-I-25 effectively inhibited the phosphorylation of IRAK4 (Figures 2B,C). However, only KT-474 was able to degrade IRAK4, thereby disrupting its scaffold function (Figures 2B,D). LPS stimulation activates IRAK4’s kinase activity, leading to downstream degradation of IRAK1 and phosphorylation of the MAPK signaling pathway. Both KT-474 and JH-I-25 were able to suppress these LPS-induced effects, indicating their capacity to interfere with IRAK4 kinase function-mediated signaling (Figures 2E–H). To determine the effect of KT-474 on the NFκB pathway, RAW 264.7 cells were pre-treated with KT-474, JH-I-25, and DMSO for 24 h and stimulated with LPS for 0, 15, 30, 60, 120 and 180 min (Figure 2I). KT-474 effectively blocked the phosphorylation of NF-κB p65 and showed inhibition of IκB-α degradation, reflecting the results of a previous study (Pereira et al., 2022). In contrast, IκB-α degradation occurred as early as 15 min in JH-I-25-treated macrophages (Figure 2I). In addition, KT-474 treatment significantly reduced LPS-induced NFκB p65 translocation into the nucleus compared to JH-I-25 treatment (Figure 2J). These results indicated that the IRAK4 degrader KT-474 alone effectively inhibited the NF-κB pathway, leading to better inhibition of TLR4 signaling pathway compared JH-I-25.

### 3.3 KT-474 suppresses the expression of inflammatory cytokines IL-6 and TNF-α

Considering that KT-474 suppresses both the MAPK and NF-κB pathways in LPS-stimulated RAW 264.7 cells, the potential inhibitory effects of KT-474 on the transcription and expression of key inflammatory cytokines IL-6 and TNF-α were analyzed. For this purpose, RAW 264.7 cells were pre-treated with various concentrations of KT-474 (20, 100, and 500 nM) or 500 nM JH-I-25 for 1 h, followed by stimulation with 0.5 μg/mL LPS for 6 h. Our results revealed that KT-474 treatment led to a dose-dependent decrease in the transcription and production of both IL-6 and TNF-α (Figures 2K–N). Notably, this inhibitory effect was more pronounced with KT-474 than with JH-I-25, even at the highest concentration of KT-474 used (500 nM). These findings further highlight the potential therapeutic advantages of KT-474 over conventional IRAK4 inhibitors in modulating inflammatory responses.

### 3.4 KT-474 degrades IRAK4 through the ubiquitin-proteasome pathway

**FIGURE 3:**
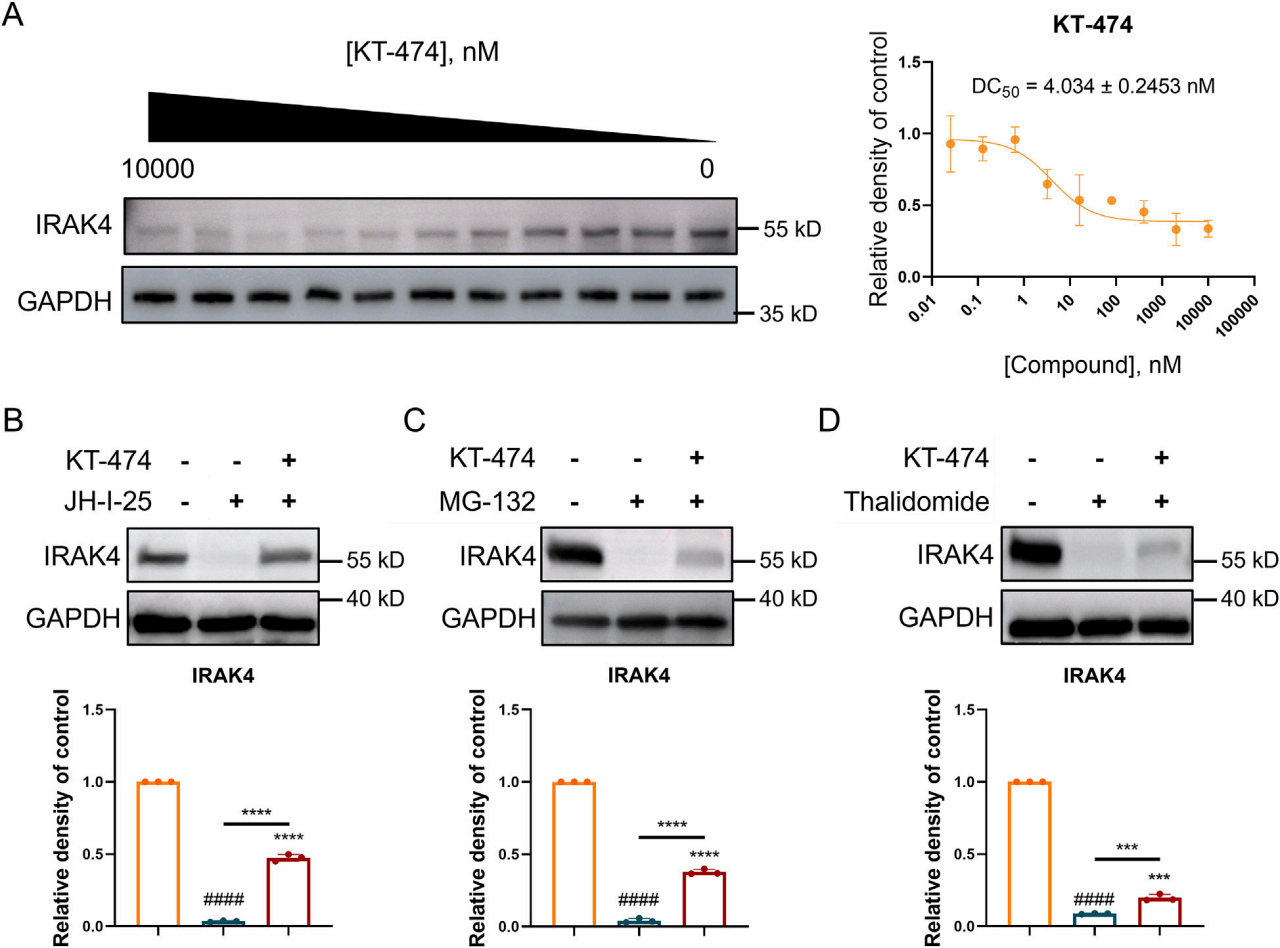
KT-474 induced the degradation of IRAK4 through the ubiquitin-proteasome system. RAW 264.7 cells were treated with KT-474 for 24 h. **(A)** Representative immunoblot images and statistical analysis of gradient KT-474 treatment RAW 264.7 cells. **(B–D)** Representative immunoblot images and statistical analysis of RAW 264.7 cells pretreated with IRAK4 inhibitor **(B)**, proteasome inhibitor **(C)** and CRBN E3 ligand **(D)** before KT-474 treatment. One-way ANOVA with Dunnett’s *post hoc* test for multiple comparisons of differences in the means of each group. n = 3-4 per group; mean ± SD; ***P < 0.001 and ****P < 0.0001; n.s. = not significant.

Considering dual role of IRAK4 in TLR signaling, straightforward inhibition might not fully harness its therapeutic potential. KT-474 emerges as a promising candidate, showing the capacity to effectively degrade IRAK4 in both *in vitro* and *in vivo* settings. Therefore, the IRAK4 degradation ability of KT-474 was further explored in RAW 264.7 cells. Our findings demonstrated that KT-474 degrades IRAK4 in a dose-dependent manner, with a DC_50_ value of 4.034 ± 0.243 nM, indicating the concentration at which 50% of IRAK4 degradation occurs (Figure 3A).

Further experiments were conducted to investigate the mechanism behind KT-474-induced IRAK4 degradation. RAW 264.7 cells were pre-treated with JH-I-25 (an IRAK4 specific inhibitor), thalidomide (a cereblon (CRBN) E3 ligand), and MG132 (a proteasome inhibitor) for 2 h before 24 h of KT-474 treatment (Figures 3B–D). The prevention of IRAK4 degradation by these inhibitors suggests that the degradation is mediated by the ubiquitin-proteasome pathway. Specifically, the involvement of the CRBN ligand indicates that KT-474 may engage the CRBN E3 ligase complex to ubiquitinate IRAK4, marking it for subsequent degradation by the proteasome.

This mechanism underscores the nuanced approach of KT-474, which does not merely inhibit IRAK4 activity but actively directs its degradation, potentially overcoming the limitations of simple inhibition strategies. The findings indicate the therapeutic potential of KT-474, leveraging the ubiquitin-proteasome system to modulate IRAK4 levels and, by extension, TLR signaling in disease contexts.

### 3.5 KT-474 attenuates LPS-induced inflammation in acute lung injury mice model

**FIGURE 4:**
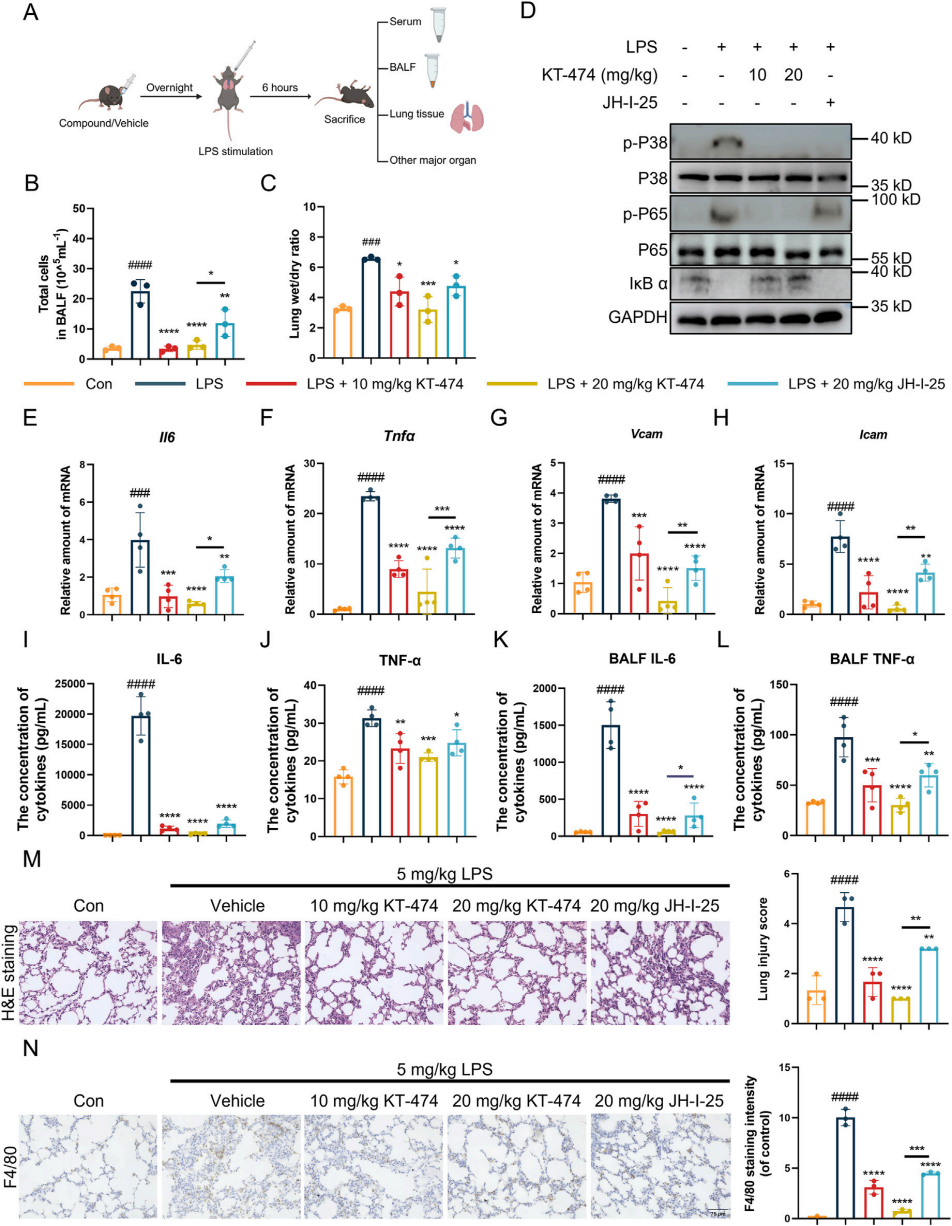
KT-474 suppresses lung injury in the LPS-induced mouse ALI model. **(A)** C57BL/6 mice were pretreated with 20 mg/kg KT-474, JH-I-25, or saline and stimulated with 5 mg/kg LPS for 6 h. **(B)** Total cell count in BALF. **(C)** Lung wet/dry ratio. **(D)** Representative immunoblots and statistical analysis of p-p38, p-NF-κB p65 and IκB-α levels in the lung tissues. **(E–H)** Real-time PCR analysis of *Il6* **(E)**, *Tnfα* **(F)**, *Vcam* **(G)** and *Icam* **(H)** levels in lung tissue. **(I–J)** IL-6 **(I)** and TNF-α **(J)** levels in serum were analyzed using enzyme-linked immunosorbent assay (ELISA). **(K,L)** IL-6 **(K)** and TNF-α **(L)** levels in BALF were analyzed using ELISA. **(M,N)** Hematoxylin and eosin **(H,E)** staining, Smith scoring analysis **(M)** and F4/80 immunohistochemical analysis by ImageJ **(N)** of the lung tissues. Scale bar, 75 μm. One-way ANOVA with Dunnett’s *post hoc* test for multiple comparisons of differences in the means of each group. n = 3-4 per group; mean ± SD; *P < 0.05, **P < 0.01, ***P < 0.001, and ****P < 0.0001; n.s. = not significant.

KT-474 has shown significant anti-inflammatory activity, exceptional kinome selectivity, and oral bioavailability (Ackerman et al., 2023; Zheng et al., 2024), and has emerged as a potential candidate for the *in vivo* LPS-induced ALI model. C57BL/6 mice were pre-treated with KT-474 at doses of 10 mg/kg and 20 mg/kg before the LPS challenge, with JH-I-25, a parent IRAK4 kinase inhibitor, serving as a positive control. Following intratracheal LPS administration (5 mg/kg), serum, BALF, lung and other major organ tissue samples were collected, as displayed in Figure 4A. Histological analysis (H&E staining) of major organs, along with serum biochemical parameters, indicated that neither KT-474 nor JH-I-25 caused significant toxicity or adverse effects in mice, supporting their safety profiles under the experimental conditions (Supplementary Figures S2A–C). LPS induced significant pulmonary injury and edema, leading to an increase in total cells in BALF (Figure 4B) and an elevated lung wet/dry weight ratio (Figure 4C). Notably, KT-474 exhibited a more potent inhibitory effect compared to JH-I-25, demonstrating its superior efficacy in mitigating lung injury. The impact of KT-474 on downstream MAPK and NF-κB activities *in vivo* was also evaluated. Immunoblotting of mouse lung tissue samples revealed that LPS challenge triggered phosphorylation of NFκB p65 and ERK while decreasing IκB-α levels (Figure 4D). JH-I-25 demonstrated significant *in vivo* MAPK inhibition, as evidenced by markedly reduced p-p38 levels; however, it did not inhibit IκB-α degradation and NFκB p65 phosphorylation. Consistent with *in vitro* findings, KT-474 showed superior inhibitory activity against NF-κB compared to the kinase inhibitor JH-I-25.

Furthermore, KT-474 can more effectively suppress the transcription of inflammatory markers (Figures 4E–H). JH-I-25 effectively reduced the expression levels of TNF-α and IL-6 in lung tissues, while KT-474 significantly diminished the LPS-induced rise in pro-inflammatory cytokines (TNFα and IL-6) in serum and BALF samples (Figures 4I–L). Histopathological examination of lung sections revealed that KT-474 attenuated the structural damage of lung tissues caused by LPS exposure (Figure 4M). Immunohistochemical analysis targeting F4/80 (macrophage marker) and MPO (neutrophil marker), were conducted to assess the impact of KT-474 on macrophage presence in the LPS-induced ALI model. The results indicated an upsurge in F4/80 and MPO immunoreactivity in the lungs of mice subjected to LPS, whereas KT-474 treatment notably reduced F4/80-positive macrophage and MPO-positive neutrophil accumulation compared to both the LPS-stimuli and the JH-I-25-treated groups (Figure 4N; Supplementary Figure S2D).

### 3.6 KT-474 suppresses LPS-induced sepsis by IRAK4 degradation

**FIGURE 5:**
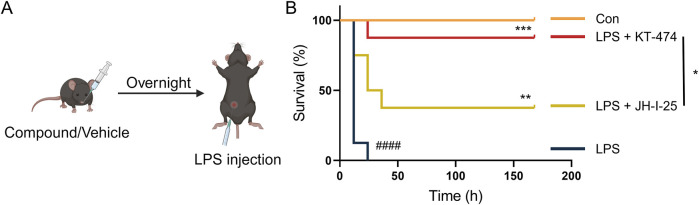
KT-474 attenuates LPS-induced inflammatory reaction and sepsis. **(A)** C57BL/6 mice were pretreated with 20 mg/kg JH-I-25, KT-474, or vehicle via intragastric injections twice a day from 12 h before LPS injection to the end of the experimental period. The survival of mice was monitored every 12 h after injection. **(B)** Survival curves of mice treated as **(A)**. Kaplan−Meier survival curves were used for analysis. n = 8 per group. *P < 0.05, **P < 0.01 and ***P < 0.001; n.s. = not significant.

ALI is a grave condition leading to approximately 30%–45% mortality in patients (Meyer et al., 2021), and the impact of KT-474 on the survival of mice with acute inflammation was evaluated. A sepsis mouse model was established by an intravenous injection of 32 mg/kg LPS. Daily intraperitoneal injections of KT-474 (at 10 mg/kg and 20 mg/kg), 20 mg/kg JH-I-25, and vehicle control were administered (Figure 5A). The 7-day observation period revealed a significantly lower mortality rate in the KT-474- and JH-I-25-treated groups compared to the sepsis model group. Notably, the effectiveness of KT-474 surpassed that of JH-I-25. According to our results, KT-474 significantly enhanced the survival of mice with acute bacterial inflammation, aligning with the observation that KT-474 counters the severe inflammatory response triggered by LPS in ALI (Figure 5B). These findings underscore the protective effects of KT-474 on LPS-induced ALI.

## 4 Discussion

IRAK4 acts as an initial kinase in the IL-1R and TLR pathways and plays a crucial role in the MyD88-dependent cascade. It is pivotal for the development of drugs targeting MyD88-dependent inflammation and cancer (Lin et al., 2025). Clinical trials have assessed numerous IRAK4 inhibitors targeting solely the kinase activity of IRAK4, but the suboptimal therapeutic outcomes may be attributed to its dual function as an active kinase and a scaffold protein. Additionally, the kinase activity of IRAK4 may lack specificity in relation to certain diseases. Thus, therapeutic strategies that focus exclusively on inhibiting the kinase activity of IRAK4 may fail to fully block its pro-inflammatory functions, highlighting the need for degraders that target both its kinase and scaffolding roles.

While numerous IRAK4 degraders have been formulated, including degrader-5 (Zhang et al., 2020), degrader-9 (Nunes et al., 2019), compound 9 (Chen et al., 2021), and KT-474, their effectiveness in treating IRAK4-associated acute inflammatory diseases remains unknown. Compared to other IRAK4 degraders such as degrader-5 (DC_50_ = 405 nM in HEK-293T cells), degrader-9 (DC_50_ = 151 nM in PBMCs), and compound 9 (IC_50_ = 4.6 μM in OCI-LY10 cells), KT-474 demonstrates significantly greater degradation potency (DC_50_ = 2 nM in OCI-LY10 cells). In addition, KT-474 offers potentially superior safety—having progressed to Phase II clinical trials (NCT06058156; NCT06028230)—and exhibits favorable pharmacokinetic properties (T_max_ = 8.0 h; C_max_ = 2.26 μg/mL in humans; bioavailability *F* = 12% in rats) (Zheng et al., 2024; Agarwal et al., 2025). These advantages collectively support the rationale for selecting KT-474 as the focus of the current study, which investigates its potential as a clinically relevant IRAK4 degrader. In RAW 264.7 cells, KT-474 demonstrated lowered IRAK4 protein concentrations (DC_50_ = 4.0 nM) and exhibited significant anti-acute inflammatory activity. Compared to traditional kinase inhibitors like JH-I-25, KT-474 exhibited superior efficacy, suggesting that complete elimination of IRAK4 may be more effective than partial inhibition of its kinase activity. Our findings underscore the influence of the IRAK4 degrader, KT-474, in the progression of LPS-induced, TLR4-driven acute inflammatory diseases.

The *in vivo* efficacy of KT-474 has also been evaluated in mouse models of TLR4-induced ALI and sepsis, where it exhibited significant anti-inflammatory activity. These findings suggest that IRAK4 degraders like KT-474 may represent a new generation of therapeutics with enhanced efficacy in treating IRAK4-dependent inflammatory diseases. Furthermore, the ability of KT-474 to target both the kinase and scaffolding functions of IRAK4 could potentially overcome the limitations of earlier inhibitors, offering a more comprehensive therapeutic approach.

Collectively, our results suggest that eliminating both the kinase and scaffolding functions of IRAK4 can enhance the therapeutic effects compared to the inhibition of kinase activity alone. The scaffold function of IRAK4 plays a pivotal role not only in TLR4-mediated ALI, but also in TLR7/8-mediated psoriasis (Cushing et al., 2017; Zheng et al., 2024) and TLR7/9-mediated SARS-CoV-2 infection (Lamphier et al., 2014; Yao et al., 2020). Given this, the therapeutic potential of KT-474 may be extended to a broader range of TLR-driven diseases, including those mediated by TLR7, TLR8, and TLR9 signaling pathways. However, as KT-474 is a PROTAC-based bifunctional molecule, it may carry certain inherent limitations associated with this class of compounds, such as potential off-target effects. Although KT-474 has been shown to selectively degrade IRAK4 *in vitro*, there remains a possibility of unknown protein interactions *in vivo* that could lead to unforeseen side effects. Therefore, future research should not only explore the therapeutic potential of KT-474 in additional disease models but also rigorously assess its safety profile in relevant patient populations to support the expansion of its clinical indications. Such investigations will be essential for determining whether KT-474 can be clinically applied to a broader spectrum of TLR-mediated diseases, ultimately supporting the expansion of its indications in clinical trials.

## Data Availability

The original contributions presented in the study are included in the article/Supplementary Material, further inquiries can be directed to the corresponding authors.

## Glossary

ALI: Acute Lung Injury
ARDS: Acute Respiratory Distress Syndrome
BALF: Bronchoalveolar Lavage Fluid
CRBN: Cereblon
ERK: Extracellular signal-Regulated Kinase
ICAM: Intercellular Cell Adhesion Molecule 1
IRAK4: Interleukin-1 Receptor-Associated Kinase 4
LPS: Lipopolysaccharide
MyD88: Myeloid Differentiation factor 88
MPO: Myeloperoxidase
NF: Nuclear Factor
PCR: polymerase chain reaction
TLR: Toll-Like Receptor
VCAM: Vascular Cell Adhesion Molecule 1
