# Supplementary figures and images for: Degradation of IRAK4 for the treatment of lipopolysaccharide-induced acute lung injury in mice
Source: Front Pharmacol. 2025 Jul 23;16:1609923. doi: 10.3389/fphar.2025.1609923 (PMC12325316; doi:10.3389/fphar.2025.1609923)

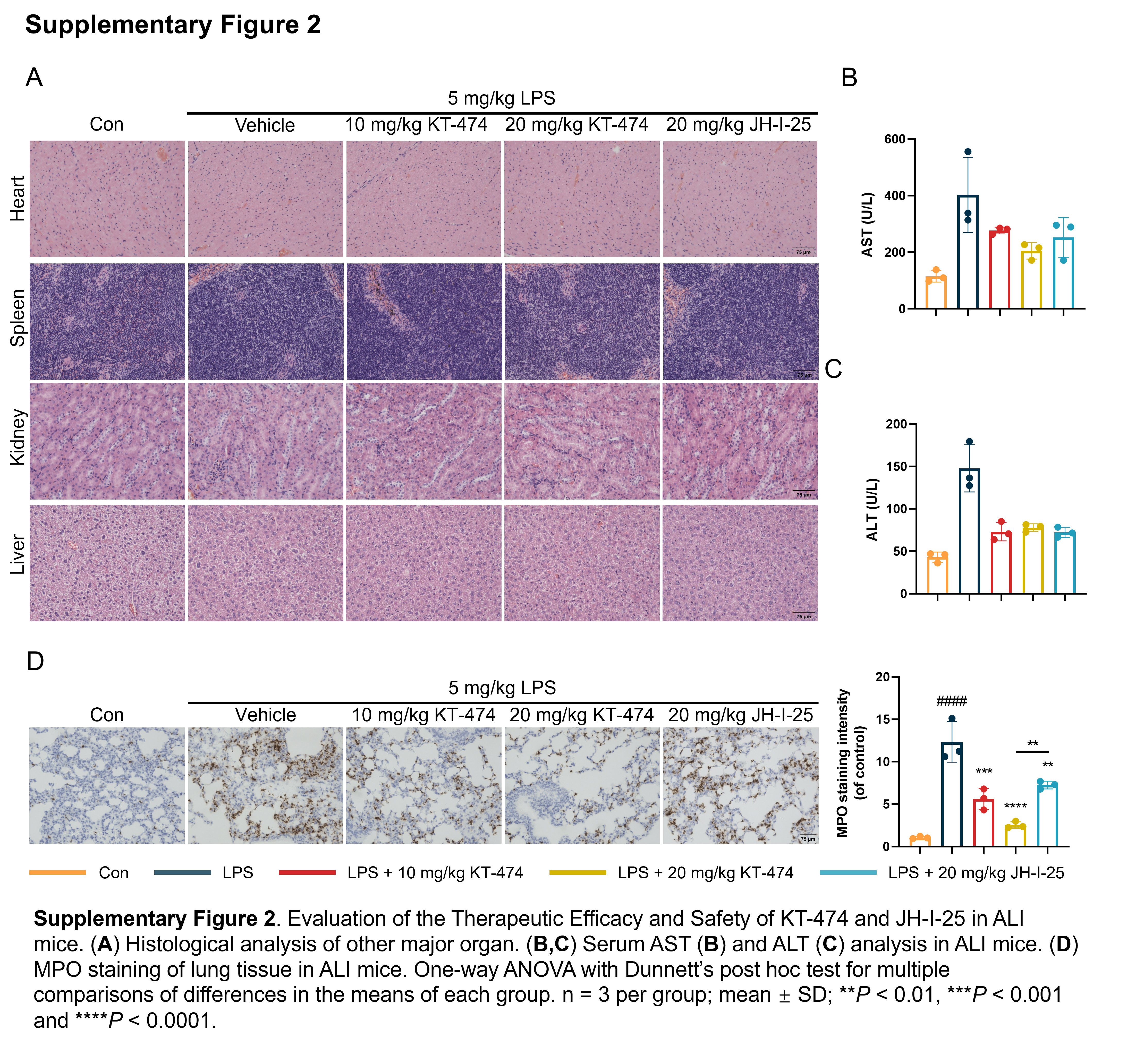

Supplement: Supplementary file 1 [file Image2.tif]

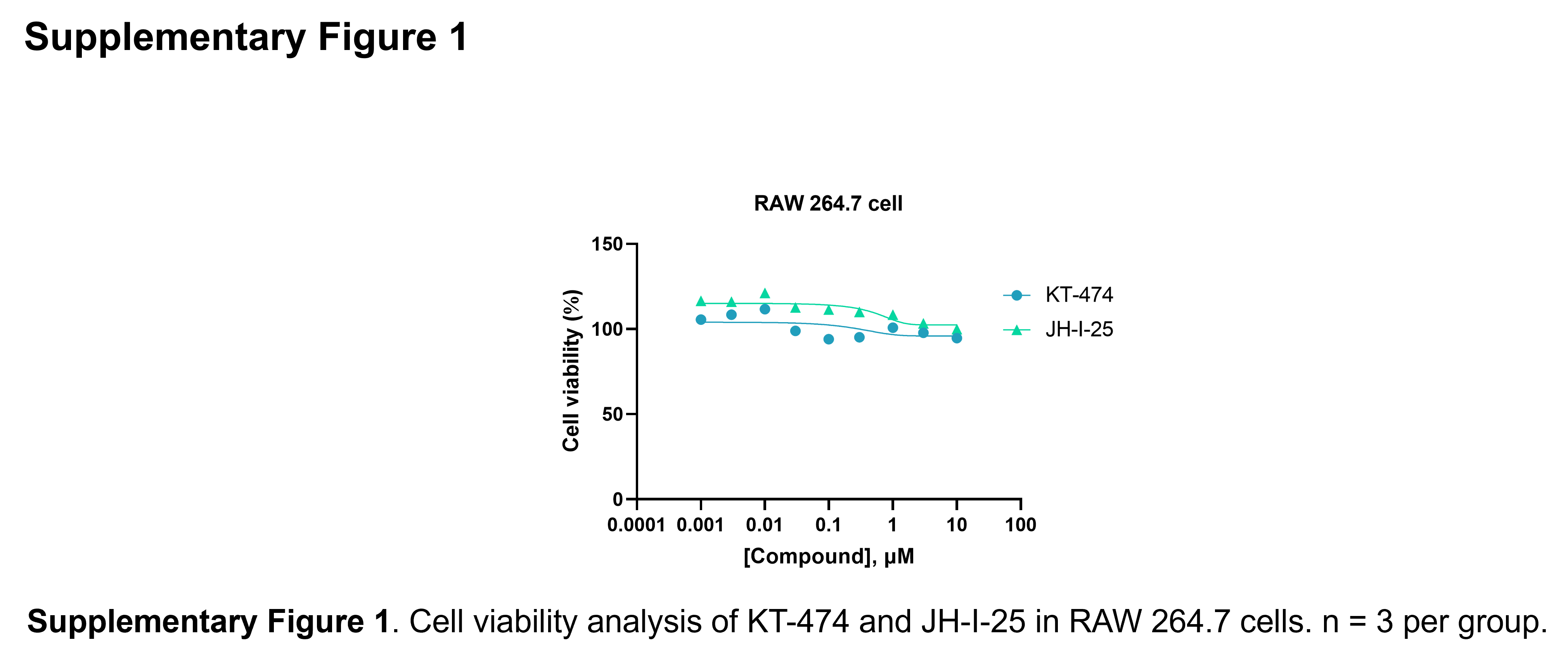

Supplement: Supplementary file 2 [file Image1.tif]
